# The role of TLR4/MyD88/NF-κB in the protective effect of ulinastatin on the intestinal mucosal barrier in mice with sepsis

**DOI:** 10.1186/s12871-023-02374-9

**Published:** 2023-12-15

**Authors:** Song Wenying, Huang Jing, Li Ying, Ding Hui

**Affiliations:** 1 Department of Anesthesiology, Shaanxi Provincial Hospital, The Third Affiliated Hospital of Xi’an JiaoTong University, Xi’an, 710068 Shaanxi Province People’s Republic of China; 2 Xi’an Medical University, Xi’an 710068, Shaanxi Province People’s Republic of China

**Keywords:** Sepsis, Ulinastatin, TLR4

## Abstract

**Objective:**

To investigate the effect of the TLR4/MyD88/NF-κB (Toll-like receptor 4/myeloid differentiation factor/nuclear factor kappa B) signalling pathway on the protective effect of ulinastatin on the intestinal mucosal barrier in mice with sepsis.

**Methods:**

A mouse model of sepsis was established by classical caecal ligation and perforation. Forty-four SPF C57BL/6 mice were randomly divided into the following four groups with 11 mice in each group: the control group (Con group), ulinastatin group (Uti group), Uti + LPS (lipopolysaccharide, LPS) group (Uti + LPS group) and LPS group. Mice in the Con group and Uti group received saline or ulinastatin injected 2 h after modelling; Mice in the Uti + LPS group received LPS injected 0 h after modelling, other procedures were the same as in the Uti group; Mice in the LPS group received LPS only. At 48 h after surgery, the levels of TNF-α (tumour necrosis factor-α, TNF-α), IL-6 (interleukin-6, IL-6) and IL-1β (interleukin-1β, IL-1β) in vein, and the expression of TLR4, MyD88 and NF-κB mRNA in small intestinal mucosa tissues using ELISA and RT‒PCR.

**Results:**

The pathological specimens showed increased inflammatory injury in the Con and LPS groups, while these injuries and changes improved in the Uti group. The scores of intestinal mucosal injury at 48 h of Uti injection were significantly lower than those of the Con group (*P* < 0.001), while the scores of intestinal mucosal injury of Uti + LPS were significantly higher than those of the Uti group (*P* = 0.044). The expression of TNF-α, IL-6 and IL-1β in the Uti decreased significantly at 48 h after surgery than that in the Con group (*P* = 0.001, *P* = 0.014, *P* = 0.004), while the expression of TNF-α, IL-6 and IL-1β in the Uti + LPS group increased significantly after surgery than that in the Uti group (*P* = 0.026, *P* = 0.040, *P* = 0.039). The expression of TLR4, MyD88 and NF-κB mRNA in the Uti group decreased significantly compared with that in the Con group (*P* = 0.001, *P* = 0.021,* P* = 0.007), while the expression of TLR4, MyD88 and NF-κB mRNA in the Uti + LPS group was higher than that in the Uti group (*P* = 0.023*, P* = 0.040*, P* = 0.045).

**Conclusion:**

These findings indicate that the protective effect of ulinastatin on the intestinal mucosal barrier against sepsis may be mediated through the TLR4/MyD88/NF-κB pathway.

## Introduction

Intestinal barrier dysfunction is an important inducing factor for the progression of sepsis. Increased intestinal permeability can potentially induce intestinal floradysbiosis. Bacterial translocation, attributed to intestinal barrier impairment, may lead to systematic infection. The intestinal epithelial barrier consists of monolayer epithelial cells and intercellular junction complexes. When sepsis occurs, the epithelial cells and intercellular junctions in the intestinal tract are vulnerable to excessive "inflammatory factor storms" [1, 2]. Therefore, revealing the proinflammatory mechanism of intestinal barrier dysfunction may be the key part of the treatment of sepsis and MODS (multiple organ dysfunction syndrome). It has been reported that sepsis is related to the activation of TLR4. The activation of TLR4 can initiate the downstream proinflammatory pathway of the MyD88/NF-κB pathway and then release a large number of inflammatory mediators, leading to intestinal injury [3].

Ulinastatin is a glycoprotein purified from the urine of healthy people. Its molecular weight is 67 kDa, and it has anti-inflammatory [4] and intestinal protective [5] effects. However, the protective mechanism of ulinastatin on the intestinal mucosa in sepsis has not been fully elucidated. The purpose of this study was to explore the molecular mechanism of the protective effect of ulinastatin on intestinal mucosal barrier function in rats with sepsis to provide a more powerful theoretical basis for ulinastatin in the treatment of sepsis.

## Materials and methods

### Animals and grouping

A total of 44 C57BL/6 male mice, aged 8–9 weeks and weighing 27 ± 2 g, were used. The room temperature was controlled at 20–25 °C, and the room was illuminated for 12 h a day. All animals ate and drank water freely. The experiment was carried out after 1 week of adaptation. This experiment was approved by the Laboratory Animal Management Committee of the Xi'an Jiao Tong University Health Science Center. The animals were studied at Shaanxi Provincial Hospital, The Third Affiliated Hospital of Xi'an Jiao Tong University Xi'an, China (ethical review number: 2020–1018, Xi'an, China). The study was carried out in obedience with the ARRIVE guidelines. All animals were treated according to the National Institutes of Health.

This study obeyed the Guidelines for the Care and Use of Laboratory Animals. Efforts were made to aggressively reduce the number of animals used and their suffering. All methods were performed in accordance with Directive 2010/63/EU in Europe and the Basel Declaration. The mice were allowed to fast and drink water ad libitum for 12 h before the experiment. The animals were randomly divided into the following 4 groups: the control group (Con group, *n* = 11), which was injected with the same volume of normal saline in tail vein 2 h after modelling; the ulinastatin group (Uti group, *n* = 11), in which 10,000 U/kg ulinastatin was injected into tail vein 2 h after modelling; the Uti + TLR4 agonist LPS group (Uti + LPS group, *n* = 11), in which LPS (5 mg/kg) was injected into tail vein 0 h after modelling and the rest was the same as the Uti group; and the TLR4 agonist LPS group (LPS group, *n* = 11), in which LPS (5 mg/kg, Sigma Company, USA) was injected into the tail vein 0 h after modelling. After 48 h of modelling, we calculated the weight loss ratio of the mice. The gut tissue samples of mice were harvested for histological study (*n* = 3, each group). Chiu’s scoring system was introduced to assess the severity of mucosal injury, it consists of 0 to 7 points, and the larger the number the more serious the injury [5]. TNF-α, IL-6 and IL-1β factor levels were measured in the tail vein blood of mice, and expression of TLR4, MyD88, and NF-κB mRNA was measured in small intestinal mucosa tissue (*n* = 8, each group).

### Reagents and drugs

LPS (Sigma Co., Ltd); ulinastatin (Sinopharm Zhunzi H19990134, 100,000 U/branch, Guangdong Tianpu Biochemical Medicine Co., Ltd.); TNF-α and IL-6 ELISA detection kits (Nanjing Jiancheng Biological Co., Ltd.); BCA protein concentration determination kits and DAB chemiluminescence kits were purchased from Beijing Huaxia Ocean Technology Co., Ltd., and TLR4, MyD88 and NF-κB antibodies were purchased from Santa Company.

### Establishment of the experimental animal model

Animals in each group fasted for 12 h before the operation and could drink water freely. Before the operation, 2% isoflurane was used for inhalation anaesthesia, routine abdominal disinfection, incision of the abdominal skin at the centre of approximately 2cm, gentle raising of the caecum, and separation of the mesentery from the distal part of the large intestine to avoid damage to mesenteric vessels. The distal 1/2 of the caecum was ligated with sterile No. 3 silk thread to avoid intestinal infarction. The needle of an aseptic 20ml syringe was used to pierce the centre of the distal caecum twice to form 4 puncture holes, and a small number of intestinal substances were gently squeezed out to prevent the puncture holes from closing. Then, the caecum was put back into the abdominal cavity, closed and sutured layer by layer.

### Haematoxylin–eosin (H-E) staining and intestinal mucosal injury scoring

The experimental mice were sacrificed 48h after the operation, and the gut tissue specimens were fixed in 10% paraformaldehyde, embedded in paraffin, cut into 5μm sections, and finally processed with haematoxylin and eosin. The modified Chiu’s scoring system was used to assess the severity of mucosal injury [5].

The modified Chiu’s scoring system was classified as follows-grade 0: normal mucosal villi; grade 1: development of edema in Gruenhagen’s space which is often accompanied by capillary congestion; grade 2: subepithelial space extension with moderate lifting of the epithelial layer from the lamina propria; grade 3: partial loss of the epithelial cells at the tip of the villus; grade 4: massive epithelial lifting along the villi sides, in addition to denuded apex; grade 5: the epithelial separation from the lamina propria has progressed from the tip towards to the base, exposing one-third to one-half of the lamina propria, moderate vasodilation and congested capillaries in lamina propria and tela submucosa; grade 6: nearly complete to complete loss of epithelium, lamina propria exposed, dilated capillaries, and an increased cellular infiltration within the lamina propria; and grade 7: disruption and disintegration of the lamina propria, presence of hemorrhage, and ulceration [5].

### Calculation of the weight change ratio of mice

The body weight of mice was recorded before and 48 h after modelling, and the weight loss ratio of mice was calculated according to the formula weight ratio = (weight after 48 h-weight before modelling)/weight before modelling.

### qRT‒PCR

Total RNA was isolated from intestinal mucosal epithelial cells at 1cm adjacent to the intestinal perforation by TRIzol (Invitrogen, USA). An M-MLV Platinum qRT‒PCR kit (Invitrogen) was used for reverse transcription and real-time quantitative PCR (RT‒qPCR). qRT‒PCR was performed on an Eppendorf Realplex 4 instrument (Eppendorf, Hamburg, Germany) at 95℃ for 4min; 35 cycles of 95℃ for 25s, 60℃ for 40s, and 72℃ for 90s; and 72℃ for 10min. Primers for the TLR4, MyD88, NF-κB and β-actin genes were obtained from Univ-bio (Shanghai, China). All the primers used in PCR are shown in Table 1:

**Table 1:** Primers for qRT‒PCR

gene	primer
TLR4	F: 50-AGAAAACTGCTCGGTCAGACG-30
	R: 50-AATGGAATCGGGGTGAAGGG-30
MyD88	F: 5′-GTTGTGTGTGTCCGACCGT-3′
	R: 5′-GTCAGAAACAACCACCACCATGC-3′
p-NF-κB	F: 5′-CCAAAGAAGGACACGACAGAATC-3′
	R: 5′-GGCAGGCTATTGCTCATCACA-3′
GAPDH	F: 5′-GACATCAAGAAGGTGGTGAAGC-3′
	R: 5′-GAAGGTGGAAGAGTGGGAGTT-3′

### Enzyme-linked immunosorbent assay for TNF-α, IL-6 and IL-1β in the tail vein

TNF-α, IL-6 and IL-1β in the tail vein were detected by an ELISA kit, followed strictly according to the instructions of the kit, and then analysed by a multifunction enzyme labelling instrument.

### Statistical analysis

All the data in this study were processed by SPSS 22.0 statistical analysis software (IBM Company, USA). Data obeying normal distribution and the measurement data were expressed as the "mean ± standard deviation" ($\overline{x}$ ± s), and median for variables that are not normally distributed. The F test was used, one-way ANOVA was used for comparisons among multiple groups, and the LSD-t test was used for pairwise comparisons between groups. *P* < 0.05 indicated that the difference was statistically significant.

## Results

### Histopathological damage in the intestine and intestinal mucosal injury score of mice in each group

**Fig. 1:**
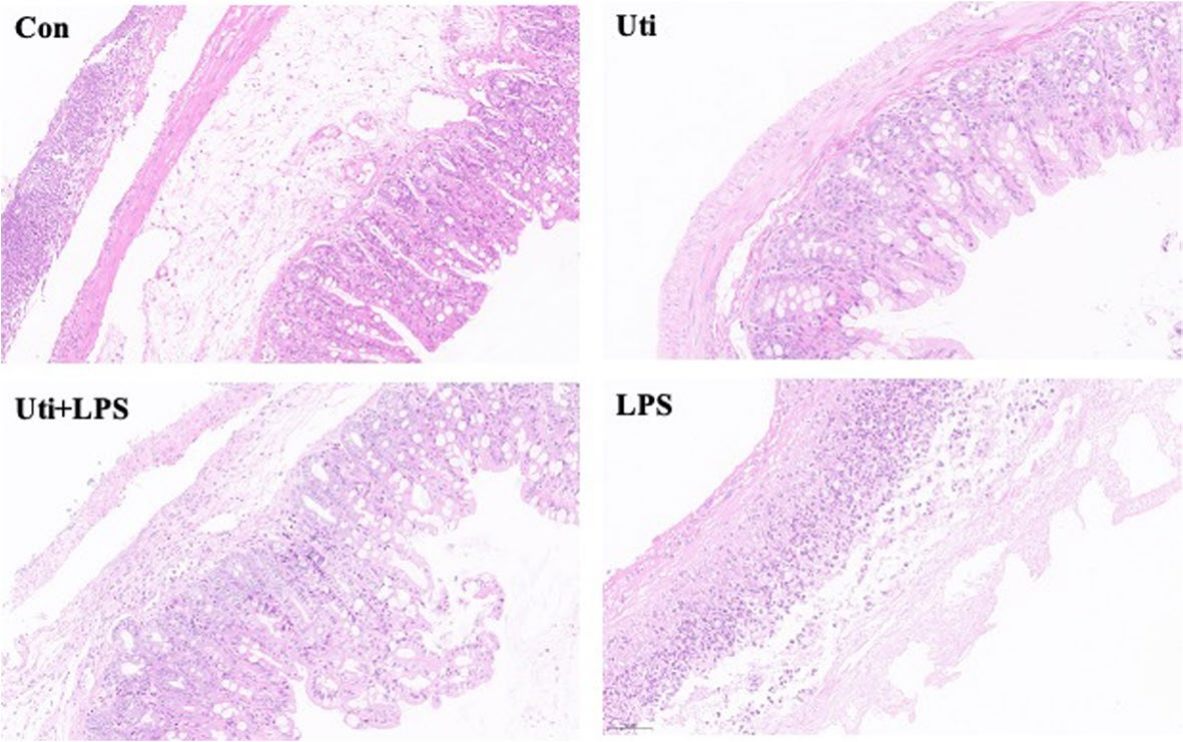
The histopathological alterations of gut tissues were measured via H-E staining in each group at 48h after the operation (*n* = 3). Scale bar = 50 μm. The ileum mucosa and submucosal stroma showed severe oedema, serious leukocyte permeation, disordered villi, and seriously damaged villi structure in the Con and LPS groups. The epithelial structure of the ileum mucosa in the Uti group was more complete, and the villi oedema was significantly improved compared with the Con group and LPS group. *Con*: Control group, *Uti*: Ulinastatin group, *Uti* + *LPS*: Uti + TLR4 agonist LPS group

**Fig. 2:**
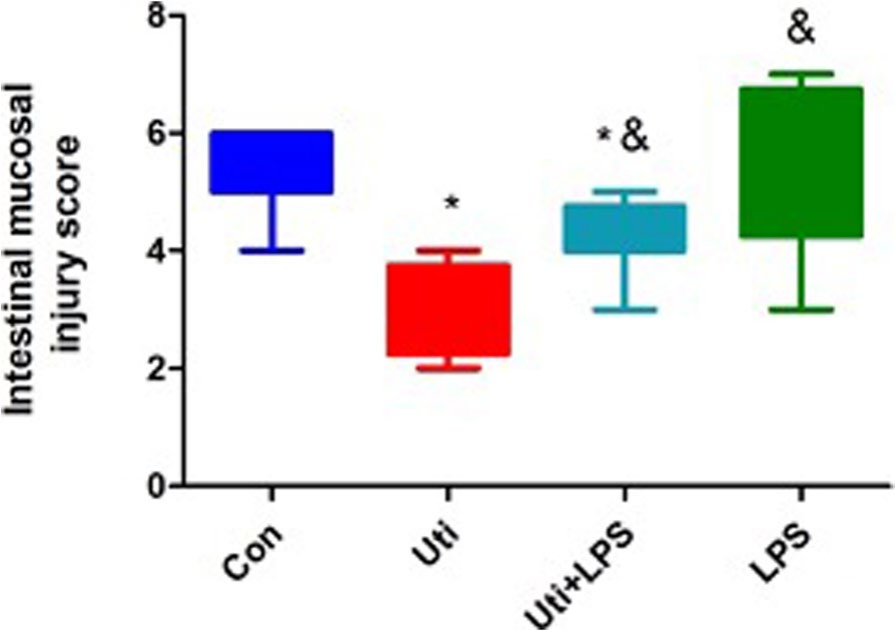
Intestinal mucosal injury score of mice in each group at 48 h after operation (*n* = 8). Data are presented as the mean ± SD. The intestinal mucosal injury scores were markedly decreased in the Uti group and Uti + LPS group in comparison with the Con group, which could be reversed by LPS. *P < 0.01 vs. Con. ^&^*P* < 0.05 vs. Uti. Con: control group; Uti: ulinastatin group; Uti + LPS: Uti + TLR4 agonist LPS group

H-E staining showed that the ileum mucosa and submucosal stroma were severely oedematous, there was serious leukocyte permeation, villi were disordered, and villi structure was seriously damaged in the Con and LPS groups (Fig. 1). These pathological alterations in the gut were significantly ameliorated by Uti administration (Fig. 1). Similarly, compared with the control group, the intestinal mucosal injury scores of mice were markedly decreased in the Uti group and Uti + LPS group (*P* = 0.000 and 0.025). The intestinal mucosal injury scores of mice in the Uti + LPS group and LPS group were significantly higher than those in the Uti + LPS group and LPS group (*P* < 0.05), and the difference was statistically significant (Fig. 2).

### Effects of ulinastatin on TNF-α, IL-6 and IL-1β in septic mice

**Fig. 3:**
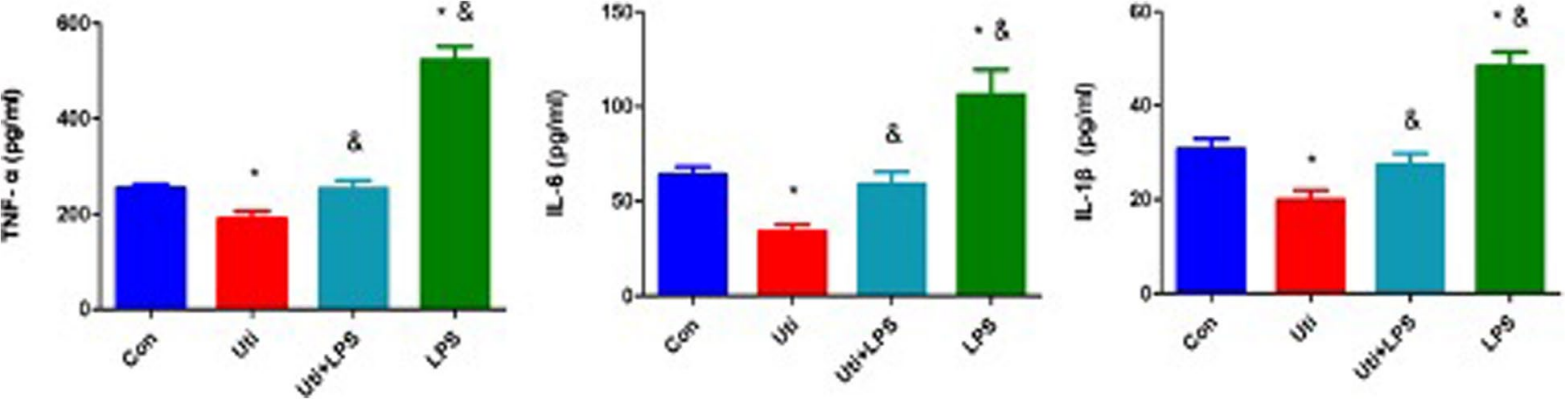
Changes in TNF-α, IL-6 and IL-1β in each group at 48 h after the operation (*n* = 8). TNF-α, IL-6 and IL-1β levels were markedly decreased in the Uti group in serum. In contrast, LPS injection increased TNF-α, IL-6 and IL-1β levels in serum compared with the Uti group. *P < 0.01 vs. Con. &*P* < 0.05 vs. Uti. *Con* Control group, *Uti* Ulinastatin group, *Uti* + *LPS* Uti + TLR4 agonist LPS group

The levels of TNF-α, IL-6 and IL-1β in the tail vein of mice were detected. The results showed that the levels of TNF-α, IL-6 and IL-1β in the Uti group were significantly lower than those in the Con group (*P* = 0. 022, *P* = 0. 014, *P* = 0. 004); the levels of TNF-α, IL-6 and IL-1β were significantly increased in the LPS group (*P* = 0. 000,* P* = 0. 001, *P* = 0. 000). Compared with those in the Uti group, the levels of TNF-α, IL-6 and IL-1β in the Uti + LPS group were significantly higher (*P* = 0. 026, *P* = 0. 040, *P* = 0. 039). The levels of TNF-α, IL-6 and IL-1β in the LPS group were significantly higher than those in the Uti group (*P* = 0. 000, *P* = 0. 000, *P* = 0. 000), and the difference was statistically significant (see Fig. 3).

### Effects of ulinastatin on the expression of TLR4, MyD88, and NF-κB mRNA in the intestinal tissue of mice

**Fig. 4:**
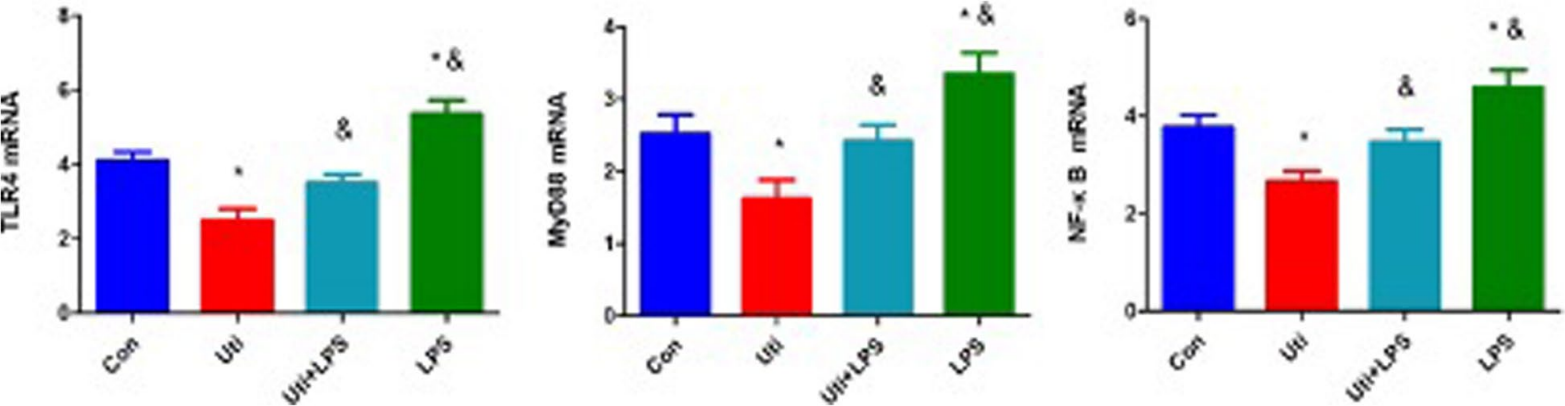
Changes in TLR4, MyD88, and NF-κB mRNA expression in intestinal tissue in each group at 48h after the operation (*n* = 8). TLR4, MyD88, and NF-κB mRNA expression were markedly decreased in the Uti group in intestinal tissue. In contrast, LPS injection increased TLR4, MyD88, and NF-κB mRNA expression compared with the Uti group. *P < 0.01 vs. Con. &*P* < 0.05 vs. Uti. *Con*: Control group, *Uti*: Ulinastatin group, *Uti* + *LPS*: Uti + TLR4 agonist LPS group

To further observe the mechanism of ulinastatin in sepsis in mice, the mRNA expression of TLR4, MyD88, and NF-κB in intestinal tissue was observed. The results showed that the expression of TLR4, MyD88 and NF-κB mRNA in the Uti group was significantly lower than that in the Con group. (*P* = 0. 001, *P* = 0. 021, *P* = 0. 007), while the expression of TLR4, MyD88 and NF-κB mRNA in the LPS group was significantly increased (*P* = 0. 008, *P* = 0. 033, *P* = 0. 042). Compared with the Uti group, the expression of TLR4, MyD88 and NF-κB mRNA in the intestinal tract of mice in the Uti + LPS group was significantly higher than that in the LPS group (*P* = 0. 023, *P* = 0. 040, *P* = 0. 045), and the expression of TLR4, MyD88, and NF-κB mRNA in the intestinal tract in the LPS group was significantly higher than that in the LPS group (*P* = 0. 000, *P* = 0. 000, *P* = 0. 000). The difference is statistically significant, as shown in Fig. 4.

## Discussion

As a common clinical critical illness, sepsis is usually caused by infection or injury and is characterized by a systemic inflammatory reaction, which is the main cause of ICU mortality [6, 7]. Köhler et al. discovered that 30% of patients with sepsis may develop septic shock in the intensive care unit [8]. During sepsis, the intestinal tract is regarded as a "motor" organ, and its mucosal epithelium has a protective barrier [9]. Once the integrity of the intestinal barrier is destroyed, a large number of bacteria and toxins enter the internal environment, which promotes sepsis to transform into septic shock or even multiple organ dysfunction syndrome (MODS) [10]. Recent studies have found that the TLR4 pathway is involved in the inflammatory state of sepsis [11] and can affect the expression of inflammatory cytokines induced by LPS in sepsis [12]. Over the years, scholars have tried a variety of new treatment techniques, but there is still a lack of effective drugs or measures for the treatment of sepsis and a lack of large-scale clinical trials. The purpose of this study was to explore the possibility of ulinastatin in the treatment of sepsis and its potential mechanism. Our results show that ulinastatin can reduce the systemic inflammatory response by inhibiting the TLR4/MyD88/NF-κB pathway in the intestine and play a protective role in septic mice.

In this study, we found that the expression of the TLR4/MyD88/NF-κB signalling pathway in the intestinal tract of septic mice was significantly increased, which aggravated the systemic inflammatory reaction. This is similar to previous studies, such as the TLR4-mediated MyD88-dependent pathway, which is involved in the immunomodulation of lipopolysaccharide in vitro and in vivo [13]. Previous studies have confirmed that the TLR4 pathway is a potential target for the treatment of neuroinflammation [14]. LPS can induce Schwann cells to produce inflammatory cytokines by activating the TLR4 pathway [12]. Our research shows that sepsis can induce a systemic inflammatory response in mice by activating the TLR4/MyD88/NF-κB signalling pathway, thus increasing the mortality of mice. Ulinastatin can significantly inhibit the expression of the TLR4/MyD88/NF-κB signalling pathway in the intestinal tract, alleviate tissue destruction and inflammatory infiltration in the intestine, and reduce the systemic inflammatory response in mice, thus reducing the mortality of mice and finally exerting a protective effect against sepsis in mice. However, TLR4 agonists can significantly increase the expression of TLR4/MyD88/NF-κB signalling, thus reversing the protective effect of ulinastatin on sepsis. Meanwhile, we also found that the weight of mice in Con and LPS groups lost significantly than those in Uti group, and we speculated that mice in Con and LPS groups with severe mucosal injury that affected their food intake and nutrition absorption, and the systemic inflammatory reaction was also serious, which further aggravates the progression of the sepsis, so the weight of mice in the severe mucosal injury group decreased significantly.

ERK is a member of mitogen-activated protein kinase (MAPK) and plays a key role in intracellular information transmission; it also participates in the transmission of extracellular stimulation signals to the nucleus, regulating gene expression and so on [15]. Previous studies have found that ERK can downregulate the expression of the MAPK pathway and relieve sepsis-related cardiac dysfunction [16]. In a study of alveolar macrophages, it was found that LPS-TLR4 activated and promoted the release of IL-1β by upregulating the MyD88/NF-κB pathway, which resulted in apoptosis of alveolar macrophages and ultimately aggravated lung injury [17]. This is similar to our research results.

Ulinastatin (UTI) is a serine protease inhibitor with multiple protective effects [18]. With its potential anti-inflammatory effect, UTI has been used to treat a variety of diseases. For example, UTI can regulate innate immunity and the proinflammatory response [19–21] and has been used to treat septicemia [22–26]. Previous studies have shown that UTI treatment can improve the survival rate of septic mice [27] and improve the intestinal injury induced by lipopolysaccharide (LPS) in mice. However, the mechanism of UTI in intestinal sepsis has not been fully elucidated. To observe the mechanism of ulinastatin in sepsis, we chose the classical caecal ligation and puncture method to establish a sepsis mouse model. In the previous treatment dose exploration, it was found that 10,000 U/kg ulinastatin injected into the tail vein 2 h after modelling had a good therapeutic effect, so the scheme was adopted at both the time point and dose. It was found that the TLR4/MyD88/NF-κB signalling pathway was involved in the anti-inflammatory mechanism of ulinastatin-induced sepsis, and this effect could be reversed by the TLR4 agonist LPS, which further confirmed that ulinastatin could reduce the inflammatory response associated with sepsis by inhibiting the expression of the TLR4/MyD88/NF-κB signalling pathway and ultimately play a role in intestinal protection.

As a common clinical critical illness, sepsis is mainly caused by the joint action of inflammatory mediators and effector cells. This study confirmed that ulinastatin can achieve intestinal mucosal barrier protection in sepsis by inhibiting TLR4/MyD88/NF-κB anti-inflammatory signal transduction and reducing inflammation-related cascade reactions and finally provides a reliable theoretical basis for the use of ulinastatin in the clinic.

## Data Availability

The raw data associate the conclusions of this manuscript will be made available by first author, without undue reservation, to any qualified researcher.
